# The importance of representation in dermatology: Addressing disparities in eczema treatment and shared decision-making

**DOI:** 10.1016/j.jdin.2023.03.011

**Published:** 2023-04-12

**Authors:** Margaux P. Games, Jules B. Lipoff

**Affiliations:** a Drexel University College of Medicine, Philadelphia, Pennsylvania; b Department of Dermatology, Lewis Katz School of Medicine, Temple University, Philadelphia, Pennsylvania

**Keywords:** dermatology, diversity, eczema, race concordance, shared decision-making (SDM)

*To the Editor:* We read with interest Foster et al’s^1^ recent article, “Factors facilitating shared decision making in eczema: met and unmet needs from the patient perspective,” which highlighted traits that patients with eczema seek from their health care providers (HCPs), with a focus on optimizing shared decision-making (SDM).

We would like to bring extra attention to one finding in Foster et al’s^1^ study: “few eczema patients perceived that the race, ethnicity, and gender of their HCP matching their own was important for SDM.” On the contrary, the data likely underestimates the importance of race, ethnicity, and gender concordance between patients and their HCPs. While the study examined many important factors related to SDM facilitation, others that could play a role were not included (eg, LGBTQ+ identification, primary language). Their data show that 16.7% of patients felt it was “very important/absolutely essential” to have patient-provider gender concordance, and “10.8%” felt it was “very important/absolutely essential” to have patient-provider race/ethnicity concordance. A trait need not be “absolutely essential” to be valuable to patients. The fact that almost half of Black patients rated race/ethnicity concordance as essential and that 49.3% did not have this need met suggests that representation among HCPs is indeed significant and insufficiently addressed.

It is well established that systemic racism impacts Black patients in the American health care system, limiting access to quality healthcare in many ways. Recruiting and encouraging more Black medical students to enter dermatology could be one straightforward approach to addressing inequities in the field. Notably, a recent study showed limited racial, ethnic, and sexual orientation diversity among students pursuing dermatology and that these students were less likely to pursue care in public health or for marginalized populations.^2^ Foster et al’s^1^ study shows the potential direct impact of recruiting a more diverse cohort of dermatologists.

The lack of Black dermatologists, representing only 3% of dermatologists (compared to 12.8% of Americans),^3^ may contribute to disparities in outcomes for Black patients with eczema due to unique dermatologic needs. Further, the underrepresentation of skin of color in medical curricula^4^ may perpetuate incomplete knowledge of the presentation of eczema on Black skin. These gaps may lead to mistrust for Black patients with eczema. For example, Black patients reported increased satisfaction with race-concordant dermatology care due to multiple factors, including knowledge of Black skin and hair.^5^ By expanding the skin color knowledge base and cultural sensitivity when evaluating and treating Black patients, dermatologists can earn greater patient trust and work toward more effective SDM relationships. Ultimately, further consideration and research should be conducted on the factors affecting minority groups with eczema and their relationship with HCPs.

## Conflicts of interest

None disclosed.
